# Serum Calcium to Phosphorous (Ca/P) Ratio Is a Simple, Inexpensive, and Accurate Tool in the Diagnosis of Primary Hyperparathyroidism

**DOI:** 10.1002/jbm4.10019

**Published:** 2017-11-02

**Authors:** Bruno Madeo, Elda Kara, Katia Cioni, Silvia Vezzani, Tommaso Trenti, Daniele Santi, Manuela Simoni, Vincenzo Rochira

**Affiliations:** ^1^ Unit of Endocrinology, Department of Internal Medicine, Endocrinology, Metabolism, and Geriatrics Azienda Ospedaliero‐Universitaria di Modena, Ospedale Civile di Baggiovara Modena Italy; ^2^ Department of Laboratory Medicine and Pathological Anatomy Azienda Unità Sanitaria Locale (USL) of Modena Modena Italy; ^3^ Department of Biomedical Metabolic and Neural Sciences University of Modena and Reggio Emilia Modena Italy; ^4^ Center for Genomic Research University of Modena and Reggio Emilia Modena Italy

**Keywords:** HYPERPARATHYROIDISM SCREENING, DIAGNOSIS, HYPERCALCEMIA, HYPOPHOSPHATEMIA, PARATHORMONE, PTH, DIAGNOSTIC VALUE

## Abstract

Primary hyperparathyroidism (PHPT) diagnosis is challenging and is based on serum calcium (Ca) and parathyroid hormone (PTH). Because serum Ca and phosphorous (P) are inversely related in PHPT, we investigated the diagnostic value of the serum Ca/P ratio in the diagnosis of PHPT. We report a single‐center, case‐controlled, retrospective study including 97 patients with documented PHPT and compared them with those of 96 controls (C). The main outcome measures were: serum PTH, 25‐OH vitamin D, Ca, P, albumin, and creatinine. Sensitivity, specificity, positive predictive value, negative predictive value, and accuracy of the serum Ca/P ratio were calculated. The results were verified using an independent, anonymous set of data extracted from a laboratory database containing over 900 million entries. A total of 35 (36.1%) PHPT patients had normocalcemic PHPT (NCHPT). Ca and PTH were significantly higher in PHPT than in C (*p* < 0.0001). P was significantly lower in PHPT than in C (*p* < 0.0001). The Ca/P ratio was significantly higher in PHPT than in C (*p* < 0.0001). Receiver‐operating characteristic (ROC) curves analyses identified a cutoff of 2.71 (3.5 if Ca and P are expressed in mg/dL) for Ca/P ratio with a sensitivity and specificity of 86% and 87%, respectively (*p* < 0.0001), confirmed by the independent, big data approach. In conclusion, Ca/P is a valuable tool for the diagnosis of PHPT and is of superior value compared to serum Ca alone, especially in NCPHT. Because Ca/P is simple, inexpensive, and easily accessible worldwide, this ratio is useful for PHPT diagnosis, especially in laboratory/medical settings relying on limited resources, such as low‐income countries. © 2017 The Authors. *JBMR Plus* is published by Wiley Periodicals, Inc. on behalf of the American Society for Bone and Mineral Research.

## Introduction

Primary hyperparathyroidism (PHPT) is the third most common endocrine disorder^1^ and is the most common cause of hypercalcemia in the outpatient setting.^2^ PHPT should be considered in any person with elevated serum calcium (Ca) levels and no clear evidence of malignancy.^3^ Similarly, PHPT should be considered in case of hypophosphatemia because the latter is present in 10% to 20% of patients with PHPT.^4^

The biochemical profile of PHPT is classically characterized by elevated serum intact parathyroid hormone (PTH) levels coupled with hypercalcemia and slight or mild hypophosphatemia (with or without hypercalciuria).^1^, ^3^ Thus, the diagnosis of PHPT is based on the combination of hypercalcemia and elevated PTH.^3^ However, not all PHPT patients exhibit this biochemical pattern. Normohormonal PHPT (NHPHPT) and normocalcemic PHPT (NCPHPT) are characterized by serum PTH close to the upper limit, but still within the normal range^5^, ^6^ and by normal serum Ca (often in the highest quartile of the normal range),^7^ respectively. Even though they are in the normal range in NHPHPT and NCPHPT, serum Ca and phosphorous (P) levels are very close to the highest and lowest limit of the normal range, respectively.^5^, ^6^, ^7^ To complicate the picture, clinical signs and symptoms are not always present, as in asymptomatic PHPT.^8^ Hence, the diagnosis of PHPT is challenging^1^, ^3^ and often delayed^2^ because of this wide spectrum of clinical and biochemical manifestations, especially in asymptomatic and NCPHPT patients.^8^ For all these reasons, physicians cannot completely rule out the diagnosis of PHPT even in presence of normal PTH or normal serum calcium.^9^

Since the 1970s the diagnosis of PHPT has dramatically increased in parallel with the introduction of measurement of serum Ca in clinical practice, because of the development of automatic chemistry analyzers.^2^, ^10^ Several strategies have been explored in the past aiming to identify tools useful to easy diagnose or screen PHPT. Among them, biochemical markers used alone (eg, urinary Ca excretion, serum P, alkaline phosphatase) or in combination were tested without success.^11^, ^12^ Serum Ca was graphically related to both serum P and PTH in an attempt to improve the diagnostic value of these measurements, but resulted only in a mirroring image of data.^13^ The chloride to phosphorous ratio^14^ and other complex metabolic tests,^15^, ^16^ such as the measurement of tubular reabsorption of phosphate^17^ and the calcium tolerance test,^12^ had no diagnostic value and never entered in the diagnostic work up of PHPT.^18^ During the last decades several other biochemical parameters, such as cyclic adenosine monophosphate renal excretion^19^, ^20^ and other complex nomograms^21^, ^22^ or diagrams, including the renal phosphorous threshold,^23^ were tested without success and none of them was introduced in routine clinical practice due to their inaccuracy, complexity, or both.

Ca and P homeostasis are directly interrelated because serum Ca interplays with serum P through the modulation of several hormones; for this reason their serum concentration is approximately inversely related.^24^, ^25^ Even in PHPT serum Ca and P are inversely related,^13^ thus the Ca to P ratio (Ca/P) might be considered a good candidate tool for the diagnosis of PHPT. Surprisingly, no data on Ca/P ratio are available in literature,^11^ despite the fact that they are very simple biochemical measurements largely available in any clinical laboratory setting.

The aim of this study is to investigate the diagnostic value of the Ca/P ratio in the diagnosis of PHPT.

## Patients and Methods

### Study design

A single‐center, retrospective, case‐control investigation, consisting of two trials, was carried out. The first trial was a retrospective analysis of patients followed in our center subdivided in two groups: patients with a certain diagnosis of PHPT (cases) and disease‐free subjects (controls). This trial was registered in http://ClinicalTrials.gov (Identifier: NCT03027349).

The second trial was an analysis of data extracted from a database of over 900 million laboratory records interrogated to verify the validity of the findings of the clinical trial.

### Trial 1: subjects

**Figure 1:**
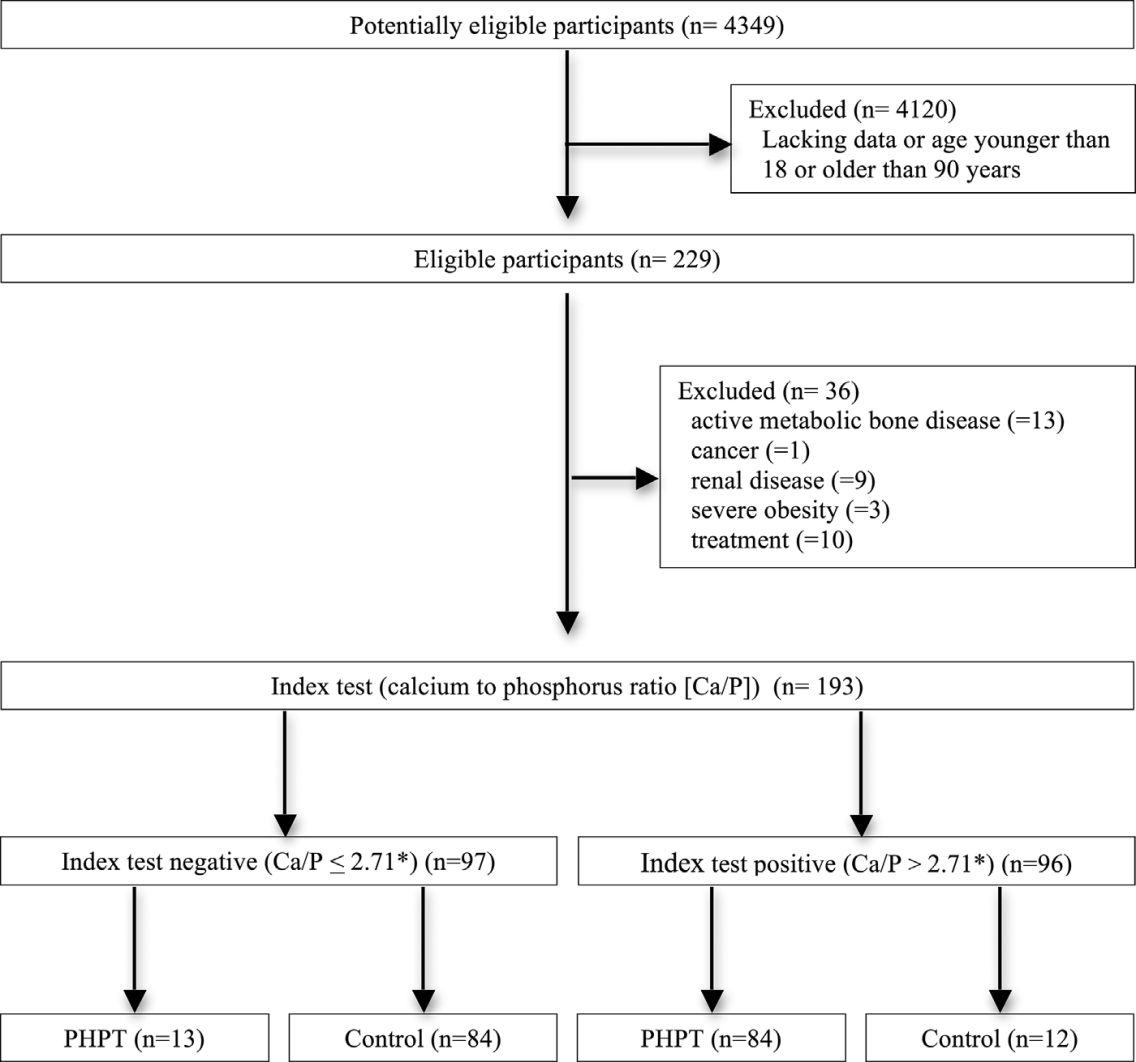
STARD flow diagram of participants through the study summarizing patients' recruitment and diagnostic accuracy of Ca/P for Trial 1. *3.5 if Ca and P are measured in mg/dL (conventional units). Ca/P = serum calcium to phosphorous ratio; PHPT: primary hyperparathyroidism; STARD = STAndards for the Reporting of Diagnostic accuracy studies.

After reviewing the record charts of all patients with PHPT who had been diagnosed at our center from 2005 to 2015, a total of 97 patients with a documented diagnosis of PHPT were included in the study (Fig. 1). The outcome measurements that had to be present in the charts for inclusion were: age, gender, serum Ca, P, PTH, 25‐OH vitamin D, and creatinine. Both patients with NCPHPT and hypercalcemic PHPT were included (Table 1, Fig. 1). As a control group, we retrospectively selected patients who attended the same center in the same time period, matched by age. Only patients with both serum PTH and Ca within the normal range and complete data set were considered.

**Table 1:** Demographic and Clinical Characteristics of All Study Participants

Subjects	*n* (%)
Controls (*n* = 96)	
Sex	
Females	44 (45.36)
Males	52 (54.63)
PHPT (*n* = 97)	
Sex	
Females	69 (71.13)
Males	28 (28.87)
PHPT pattern	
Hypercalcemic	62 (63.9)
NCPHPT	35 (36.1)
Morphological evidence of enlarged parathyroid	
Not localized enlarged parathyroid	2 (2.1)
Localized enlarged parathyroid	95 (97.9)
Localization according to each diagnostic procedure	
US (*n* = 97^a^)	89 (91.7)
CT (*n* = 12^a^)	9 (9.2)
FNAB	
Cytological evidence (*n* = 50^a^)	11 (11.3)
PTH in washing fluid of fine needle aspiration biopsy (*n* = 48^a^)	36 (37.1)
Scintigraphy (*n* = 57^a^)	35 (36.1)
Localization in patients who underwent surgery	56 (57.8)^b^
Histological evidence at parathyroidectomy	54 (55.7)
No evidence of enlarged parathyroidectomy	2 (2.1)
Localization according to combined diagnostic procedures (radiological and cytological) in not‐operated patients	41 (42.3)
By all 4 diagnostic procedures (US+FNAB+Scintigraphy+CT)	1 (1)^c^
By 3 of the 4 diagnostic procedures	4 (4.1)^c^
US+FNAB+Scintigraphy	3 (3.1)^c^
US+FNAB+CT	1 (1)^c^
By 2 of the 4 diagnostic procedures	22 (22.7)^c^
US+FNAB	8 (8.3)^c^
US+Scintigraphy	12 (12.4)^c^
US+CT	2 (2.1)^c^
By 1 of the 4 diagnostic procedures	14 (14.4)^c^
US	13 (13.4)^c^
Scintigraphy	1 (1)^c^

Diagnostic tools used to clinical confirm the diagnosis of PHPT are summarized. Of the 41 not‐operated patients, 14 did not have clinical indications for surgery, 8 were not operated because of high risk related to surgical procedures, 8 patients refused to undergo surgery, and 11 patients were still waiting for surgery at the time of data collection.

PHPT = primary hyperparathyroidism; NCPHPT = normocalcemic PHPT; US = ultrasound; CT = computed tomography; FNAB = fine needle aspiration; PTH = parathyroid hormone.

^a^ Number of patients who underwent the diagnostic procedure.

^b^ In our series, 56 of the 97 patients with PHPT underwent surgery, while the remaining 41 were not operated. All 56 patients who underwent surgery had clinical indications to surgery, according to criteria established by guidelines on PHPT.^(8)^

^c^ Percentage refers only to operated patients (*n* = 41).

Exclusion criteria for both cases and controls were as follows: age younger than 18 years or older than 90 years; severe renal and liver diseases (ie, glomerular filtration rate [GFR] <30 mL/min); hyperparathyroidism secondary to vitamin D deficiency; active metabolic bone disease (eg, Paget's disease of the bone, osteomalacia, rickets); any type of cancer; malnutrition; severe obesity (BMI >40 kg/m^2^); a history of gastrointestinal malabsorption; sarcoidosis; hypercortisolism, diabetes insipidus, hyperthyroidism, pseudohypoparathyroidism; familial hypocalciuric hypercalcemia [FHH]; hypophosphatemia not due to PHPT (eg, genetic causes); and treatment with steroids, active forms of vitamin D (calcitriol, ergocalciferol, etc.), thiazides, phosphate binders, lithium, cinacalcet, bisphosphonates, and denosumab.

Demographic characteristics of patients and controls are summarized in Table 1.

### Trial 2: data mining

All examinations of PTH, Ca, and P consecutively performed from January 2010 to December 2016 in our central laboratory of the Department of Clinical Pathology Azienda USL of Modena, Italy, were included in a large database. The database was refined considering only “datasets” including the simultaneous determination of Ca, P, PTH, and creatinine on the same occasion and belonging to subjects between 18 and 90 years of age with normal renal function, assessed by calculating GFR using the following Modification of Diet in Renal Disease (MDRD) formula: GFR = 186 × (serum creatinine)^−1.154^ × (age)^−0.203^ × (1.210 if black) × (0.742 if female). Only datasets from subjects with GFR >30 mL/min were retained for final analysis. Datasets suggesting hypoparathyroidism (PTH and/or Ca below the 5th centile) were excluded from the analysis. The remaining datasets were divided into: (i) PHPT (both PTH and Ca serum levels above the 95th centile), and (ii) controls, defined by PTH and Ca serum levels between the 5th and the 95th centile.

### Laboratory analyses

Serum Ca and P were detected using Beckman Coulter AU 680 (Beckman Coulter Italy, Cassina de' Pecchi, Milan, Italy) device by the colorimetric photometric methods based on Arsenazo III and molybdate, respectively. For both sexes, the normal reference ranges for serum Ca and P were 2.12 to 2.65 mmol/L (8.5 to 10.6 mg/dL) and 0.81 to 1.65 mmol/L (2.5 to 5.1 mg/dL), respectively.

Serum Intact PTH was determined by a Beckman Coulter UniCel DxI 600 Synchron Access (Beckman Coulter Italy). The normal reference range for serum PTH is 15 to 88 ng/L, with an intraassay variability coefficient of 5%.

Serum 25OH‐Vitamin D was measured by chemiluminescence with the LIASON XL 1,25OH‐Vitamin D assay (DiaSorin, Stillwater, MN, USA). Normal reference range is 74.9 to 249.6 nmol/L and the intraassay and interassay variability coefficient was <10%.

In order to obtain an estimation of free Ca (corrected Ca) levels we applied the following formula to all patients for which serum albumin was available in the record chart: corrected total calcium (mmol/L) = total serum calcium (mmol/L) + 0.02[40–serum albumin (g/L)].^3^

### Ethics

The Institutional Review Board of Azienda USL of Modena approved the study (Protocol n. 0032443/15).

### Statistical analysis

All data of trial 1 are shown as median and minimum to maximum and the nonparametric Mann‐Whitney *U* test was used for group comparisons because variables were not normally distributed at the Kolmogorov‐Smirnov test.

Sensitivity, specificity, positive predictive value (PPV), negative predictive value (NPV), and accuracy were evaluated.^26^

The statistical analyses of the datasets of trial 2 were performed first evaluating the variables distribution by the Shapiro‐Wilk test. The normal range for PTH, Ca, and P was obtained calculating the 95% confidence interval (CI). The Ca/P ratio was calculated and the difference between PHPT and controls was evaluated by ANOVA univariate or Mann Whitney test for normally and not‐normally distributed variables, respectively.

In both trials, the diagnostic accuracy of Ca/P was investigated using receiver‐operating characteristic (ROC) curves in order to define cutoff points that better identify affected patients according to their biochemical profile. ROC cutoffs were calculated by the Youden's index through the identification of the best pair of sensitivity and specificity.

Statistical analyses were performed using the Statistical Package for the Social Sciences' (SPSS) software for Windows (version 16.0; SPSS Inc, Chicago, IL, USA) and Sigma Plot (version 11.00; Systat Software Inc, San Jose, CA, USA). For all comparisons, *p* < 0.05 was considered statistically significant.

## Results

### Trial 1

Clinical and demographic characteristics of both PHPT patients and controls together with the diagnostic procedure(s) used for the localization of enlarged parathyroid glands in PHPT patients are summarized in Table 1.

Serum Ca was significantly higher in PHPT patients than in controls (*p* < 0.0001) (Table 2). Conversely, serum P was significantly lower in PHPT patients than in controls (*p* < 0.0001) (Table 2). Serum albumin obtained from the record charts allowed the calculation of corrected Ca in 73 PHPT patients (75.25%) and 67 controls (69.8%). Serum corrected Ca was significantly higher in PHPT patients than in controls (*p* < 0.0001) (Table 2). Both serum Ca/P and corrected Ca/P were significantly higher in patients with PHPT than in controls (*p* < 0.0001) (Table 2). As expected, serum PTH was higher in patients with PHPT than in controls (*p* < 0.0001). Age and serum creatinine did not differ between the two groups (Table 2).

**Table 2:** Age, Biochemical, and Hormonal Differences Between Patients With PHPT and Controls

	Normal range	PHPT (*n* = 97)	Controls (*n* = 96)	*p*
Age (years)	18–90^a^	63 (23–90)	58.5 (20–89)	0.34
Serum Ca (mmol/L)	2.12–2.65	2.75 (2.35–3.87)	2.35 (2.07–2.55)	<0.0001
Serum P (mmol/L)	0.81–1.65	0.77 (0.45–1.26)	1.14 (0.68–1.45)	<0.0001
Ca/P	–	3.56 (2.03–6.81)	2.09 (1.55–3.56)	<0.0001
Serum PTH (ng/L)	15–88	135.2 (57.6–1748)	32.1 (14–80.7)	<0.0001
Serum 25‐OH vitamin D (nmol/L)	74.9–249.6	40.7 (10–110)	50.8 (10–103)	0.008
Serum creatinine (mmol/L)	44.2–123.8	70.7 (44.2–132.6)	70.7 (44.2–150.2)	0.454
Serum albumin (g/L)^b^	35–50	43 (27–48)	41 (26–50)	0.01
Serum corrected Ca (mmol/L)^b^	–	2.75 (2.32–3.97)	2.3 (2.1–2.67)	<0.0001
Corrected Ca/P ratio^b^	–	3.56 (2.09–6.81)	2.01 (1.63–3.41)	<0.0001

Measurements are expressed as median (minimum–maximum).

PHPT = primary hyperparathyroidism; Ca = serum calcium; P = serum phosphorous; Ca/P = serum calcium to phosphorous ratio.

^a^ Age range for enrolment in this study.

^b^ Available in 73 PHPT patients (75.25%) and 67 controls (69.8%).

**Figure 2:**
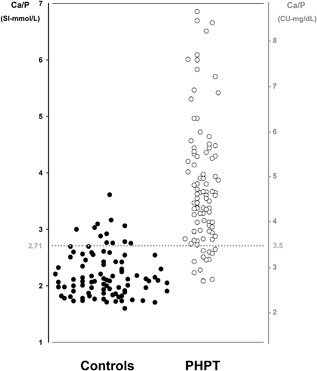
Distribution of Ca/P values between controls and PHPT patients according to the Ca/P threshold of 2.71 (3.5 in conventional units with Ca and P measured in mg/dL). Ca/P = serum calcium to phosphorous ratio; SI = International System of Units (Ca and P measured in mmol/L); CU = conventional units (Ca and P measured in mg/dL); PHPT = primary hyperparathyroidism.

**Figure 3:**
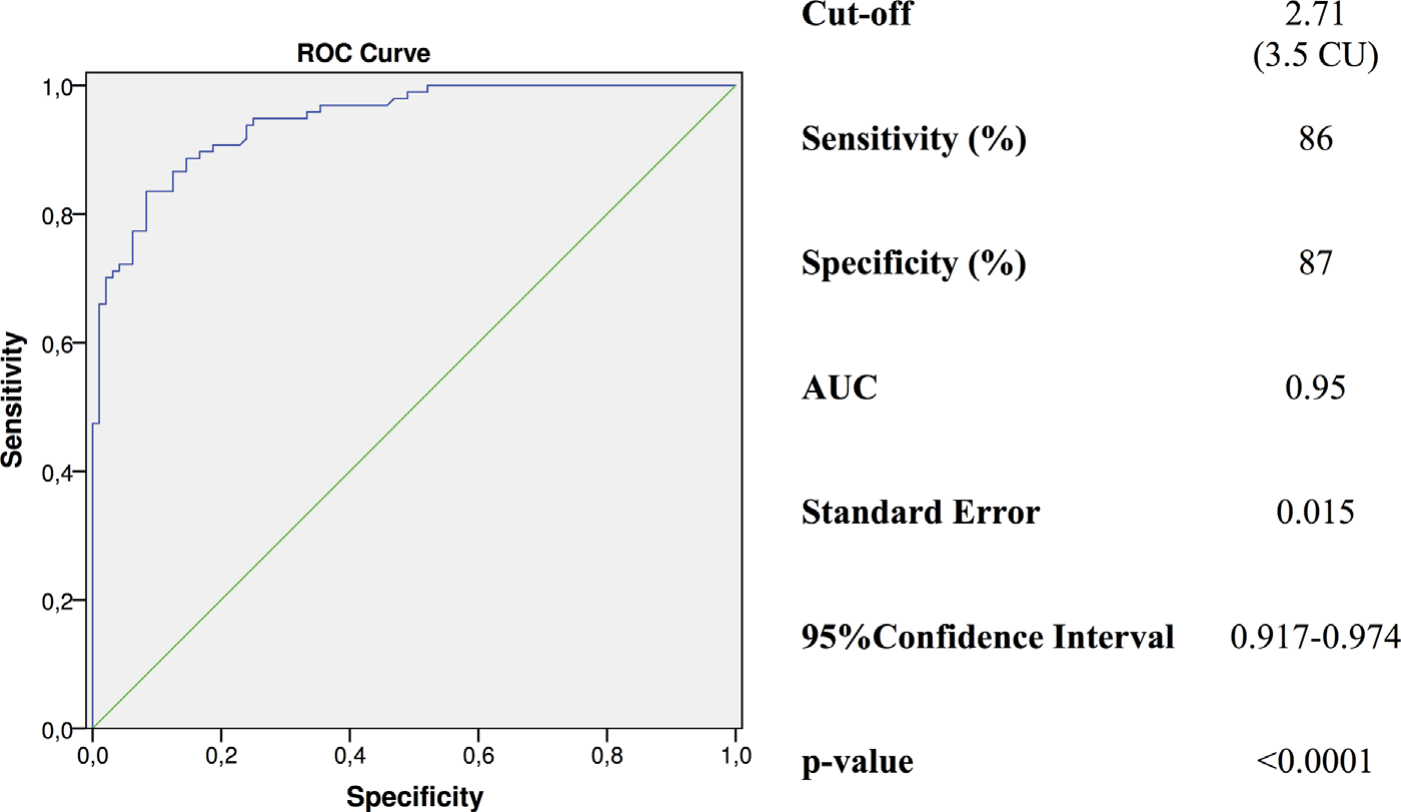
ROC curve analysis for Ca/P together with cutoff, sensitivity, specificity, area under the curve, standard error, and 95% confidence interval values for Trial 1. CU = conventional units (Ca and P measured in mg/dL); AUC = area under the curve.

The diagnostic value of diagnostic parameters included in the protocol and their ability to diagnose PHPT are summarized in Table 3. The Ca/P was able to correctly identify 84 of the 97 PHPT patients when a threshold of 2.71 (3.5 if Ca and P are measured in mg/dL) was used, while only 12 of the 96 controls were erroneously diagnosed as having PHPT (Figs. 1 and 2). The Ca/P threshold of 2.71, obtained by ROC curve analysis, performed at best for the highest sensitivity and specificity (Fig. 3). With this cutoff, the diagnostic power of Ca/P in identifying patients with PHPT was better than that of either serum Ca or corrected Ca and of all other diagnostic procedures, with exception of serum PTH, as expected (Table 3). Accordingly, sensitivity, NPV, and accuracy of Ca/P were greater than that of serum Ca and corrected Ca and other diagnostic procedures (Table 3). The best specificity was obtained by serum Ca and corrected Ca and PTH, as expected (Table 3). Concerning serum PTH, a normal serum PTH was a prerequisite for the enrolment of controls, thus overestimating its diagnostic value in this setting.

**Table 3:** Diagnostic Value of Several Parameters for the Diagnosis of PHPT in the Entire Cohort

	Cutoff	Sensitivity	Specificity	PPV	NPV	Accuracy
Ca/P	2.71 (3.5 CU)	86	87	87	86	87
Corrected Ca/P ratio	2.71 (3.5 CU)	89	91	91	88	90
Serum PTH	88	86	100	100	87	93
Serum Ca	2.65 (10.6 CU)	64	100	100	73	82
Serum corrected Ca	2.65 (10.6 CU)	63	99	98	71	80
Serum P	2.5	53	93	88	66	72
Combined Ca/P and/or serum Ca^a^	2.71/2.65 (3.5/10.6 CU)	90	87	88	89	93

Values are expressed as percentages.

PHPT = primary hyperparathyroidism; Ca = calcium; P = phosphorous; Ca/P = serum calcium to phosphorous ratio; CU = conventional units (Ca and P measured in mg/dL); Sensitivity = number of true positives divided by the number of true positives plus the number of false negatives; Specificity = number of true negatives divided by the number of true negatives plus the number of false positives; PPV = positive predictive value: number of true positives divided by the number of true positives plus the number of false positives; NPV = negative predictive value: number of true negatives divided by the number of true negatives plus the number of false negatives; Accuracy = number of true positives plus the number of true negatives divided by the number of true positives plus the number of true negatives plus the number of false positives plus the number of false negatives.

^a^ Combined Ca/P and/or serum Ca was considered positive when at least one of the two biochemical parameters was above the cutoff.

The combined use of Ca/P and serum Ca (which was considered positive when at least one of the two biochemical parameters was above the cutoff) had the best sensitivity with a concomitant, very high specificity together with PPV, NPV, and accuracy higher than Ca/P alone (Table 3).

The results obtained by considering only the subgroup of patients with NCPHPT were similar to those obtained considering all the subjects, with the exception of a lower diagnostic performance of both serum Ca and corrected Ca, as expected (Table 4). Among NCPHPT, the Ca/P was able to identify 25 out of the 35 patients that could not be identified by serum Ca alone or by corrected Ca alone.

**Table 4:** Diagnostic Value of Several Parameters for the Diagnosis of PHPT in the NHPHPT Cohort

	Cutoff	Sensitivity	Specificity	PPV	NPV	Accuracy
Ca/P	2.71 (3.5 CU)	71	88	68	89	83
Corrected Ca/P ratio	2.71 (3.5 CU)	67	91	73	88	85
Serum PTH	88	89	100	100	96	97
Serum Ca	2.65 (10.6 CU)	0	100	0	73	73
Serum corrected Ca	2.65 (10.6 CU)	13	99	75	76	76
Serum P	2.5	26	93	56	77	75

Values are expressed as percentages.

PHPT = primary hyperparathyroidism; NCPHPT = normocalcemic hyperparathyroidism; Ca = calcium; P = phosphorous; Ca/P = serum calcium to phosphorous ratio; CU = conventional units (Ca and P measured in mg/dL); Sensitivity = number of true positives divided by the number of true positives plus the number of false negatives; Specificity = number of true negatives divided by the number of true negatives plus the number of false positives; PPV = positive predictive value: number of true positives divided by the number of true positives plus the number of false positives; NPV = negative predictive value: number of true negatives divided by the number of true negatives plus the number of false negatives; Accuracy = number of true positives plus the number of true negatives divided by the number of true positives plus the number of true negatives plus the number of false positives plus the number of false negatives.

### Trial 2

**Figure 4:**
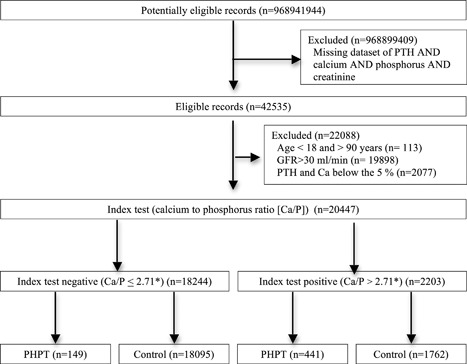
STARD flow diagram of participants through the study summarizing patients' recruitment and diagnostic accuracy of Ca/P for Trial 2. *3.5 if Ca and P are measured in mg/dL (conventional units). GFR = glomerular filtration rate; PTH = parathyroid hormone; Ca = serum calcium; Ca/P = serum calcium to phosphorous ratio; PHPT = primary hyperparathyroidism; STARD = STAndards for the Reporting of Diagnostic accuracy studies.

The starting database enclosed 968,941,944 records, of which 42,535 datasets had complete data required (PTH, Ca, P, and creatinine) as inclusion/exclusion criteria (Fig. 4). A total of 19,898 records were excluded because of a GFR<30 mL/min and 113 for age below 18 years or above 90 years. A total of 22,524 datasets remained and were used to evaluate data distribution. Ca and P were normally distributed with 95% CI from 2.1 to 2.5 mmol/L for Ca and from 0.8 to 1.4 mmol/L for P. PTH showed a leptokurtic distribution (asymmetry 2.11+0.02), thus a logarithmic transformation was applied to obtain a normal distribution with a 95% CI between 17.5 and 78.0 ng/L. A total of 2077 records showed PTH or Ca or both under the 5th centile of distribution, possibly representing samples from patients with hypoparathyroidism. The latter were excluded and the final analysis was performed on 20,447 datasets (Fig. 4). Among these, 590 datasets (2.9%) were above the 95th centile for both PTH and Ca, possibly representing the group of PHPT records (Fig. 4).

The Ca/P was able to correctly identify 441 of the 590 (74.7%) PHPT records when a threshold of 2.71 (3.5 if Ca and P are measured in mg/dL) was used, while only 1762 of the 19857 (8.8%) controls records were erroneously diagnosed as having PHPT (Fig. 4).

The Ca/P ratio was significantly higher in PHPT records (3.18; 95% CI, 1.48 to 7.55) compared to controls records (2.13; 95% CI, 1.03 to 8.58) (*p* < 0.001).

**Figure 5:**
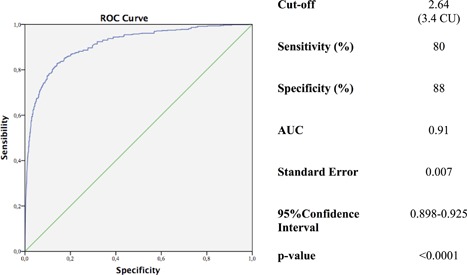
ROC curve analysis for Ca/P together with cutoff, sensitivity, specificity, area under the curve, standard error, and 95% confidence interval values for Trial 2. CU = conventional units (Ca and P measured in mg/dL); AUC = area under the curve.

By ROC curve analysis a threshold of 2.64 (3.4 if Ca and P are measured in mg/dL) for the Ca/P ratio was indicated by the best pair of values for the sensitivity and specificity (Fig. 5). With this cutoff, the diagnostic power of Ca/P ratio showed a sensitivity of 80% and a specificity of 88% (Fig. 5).

## Discussion

This study demonstrates, for the first time, that the diagnostic power of the serum Ca/P ratio is of superior value, compared to serum Ca (or corrected Ca) alone, for the diagnosis of PHPT, especially in the subgroup of NCPHPT patients. In fact, a Ca/P value above 2.71 (3.5 if Ca and P are measured in mg/dL) resulted to be an accurate, highly sensitive, and highly specific tool for the diagnosis of PHPT. More importantly, both sensitivity and accuracy further increases if serum Ca and Ca/P are used in combination (Table 3). These results were obtained from a retrospective clinical trial and were confirmed by a second, independent analysis, performed using a very large database extracted from the entirety of laboratory diagnostic determinations of a large routine laboratory, consisting of over 900 million data records. Therefore, using two different experimental approaches, we conclude that the serum Ca/P ratio should be considered a simple and very powerful diagnostic tool of PHPT.

That the Ca/P has a good diagnostic value in detecting PHPT is not surprising in view of (i) the inverse relationship between serum Ca and P in physiological conditions (ie, healthy, control subjects)^24^, ^25^; (ii) the almost constantly divergent pattern of serum Ca (increasing) and P (decreasing) in PHPT^1^, ^3^, ^13^, ^24^; (iii) the unchanged or increased serum P in case of hypercalcemia not due to PHPT^27^; and (iv) the rarity of hypophosphatemia not due to PHPT.^4^, ^28^ In the case of malignant hypercalcemia due to PTH‐like peptides, Ca/P is expected not to distinguish it from PHPT, because serum P tends to be low, but the measurement of PTH can help differentiating the two forms.^27^

This study proves that Ca/P is a highly reliable tool, especially in NCPHPT patients, who are not clearly identified as having PHPT by the measurement of serum Ca (or corrected Ca) alone.^7^, ^29^ In these patients Ca/P had the best diagnostic value in terms of sensitivity and accuracy compared to other diagnostic tools such as serum Ca and corrected Ca. Accordingly, both serum Ca and ionized Ca alone fails to recognize some cases of PHPT with normal (or fluctuating around the upper normal limit) serum Ca,^29^, ^30^ the diagnosis of NCPHPT requiring the demonstration of elevated serum PTH.^7^ Serum P alone was unreliable in the diagnosis of PHPT, in accordance with all previous studies that evaluated this parameter alone and never in combination with Ca.^11^, ^12^, ^15^, ^18^ Thus, Ca/P is more accurate than either serum P or Ca alone, avoiding false‐negative diagnoses, as suggested by the 25 of 35 (71%) patients recognized as having NCPHPT in this study. The timely diagnosis of PHPT is useful for preventing the development of comorbidities related to PHPT,^31^, ^32^, ^33^ as well as the evolution toward symptomatic, overt PHPT,^34^ the latter occurring in a relevant percentage (about 40%) of cases.^8^

In clinical practice, the use of Ca/P has the potential of becoming a useful tool for the diagnosis of PHPT and might become an inexpensive, first‐line examination in the workup of PHPT, with serum PTH as a successive measurement, useful to confirm the diagnosis of PHPT when Ca/P is above 2.71 (3.5 if Ca and P are measured in mg/dL). It is well known that serum P alone is unreliable for diagnosing PHPT, and it is currently measured neither in clinical^18^ nor in research settings, with the exception of few studies,^35^ in accordance to clinical guidelines recommendations.^8^, ^36^, ^37^, ^38^ Here we suggest that serum P should be measured together with Ca allowing calculating Ca/P to be inexpensive,^39^ and not requiring further technical equipment/skills beyond those already available for serum Ca measurement. In addition, Ca/P could be considered a good candidate for PHPT screening, especially if integrated with the serum Ca value.

This study consists of a retrospective clinical trial and a retrospective data mining and statistical analysis of a large set of data from a high‐throughput routine laboratory. The results of both trials are concordant in identifying the serum Ca/P as a powerful diagnostic tool for PHPT. Both approaches have strengths and limitations. The main strengths are (i) the simple and straightforward design of the retrospective clinical trial in which all patients with PHPT had the biochemical diagnosis confirmed by documented enlarged parathyroid/s at histological verification (55.7%) or by imaging (Table 1); and (ii) the very large data set and rigorous statistical approach to the information deriving from it in the second trial. The retrospective nature represents the main limit of this study, but assures a correct assignment to control or PHPT groups in the clinical study (Trial 1). The results refer to a single center and large, long‐term, prospective, multicenter studies on larger sample size are required to confirm and validate our findings. Other limits of this study are that patients and controls were not very well matched on gender in Trial 1, and the big data approach does not allow having a certain diagnosis for each patient because that patient's record charts are unavailable because blood samples come from several hospitals (5) and several points where blood samples are obtained from outpatients. Furthermore, the lack of a group of patients with FHH limits the use of serum Ca/P in the differential diagnosis between PHPT and FHH and we do not know its diagnostic value in this setting.

In conclusion, this study shows, for the first time and using two independent approaches, that the serum Ca/P ratio is a valuable, highly sensitive, highly specific tool for the diagnosis of PHPT and has the potential of becoming a good, cost‐effective, first‐line examination in the clinical workup of PHPT diagnosis and screening, especially in combination with serum Ca. This might be particularly useful especially in laboratory/medical settings relying on limited resources, in which PTH determination is difficult or not possible. In this regard the use of the Ca/P ratio for the diagnosis of PHPT can be extended to every clinical and laboratory setting worldwide, including developing countries, reducing health inequalities^40^ due to limited access to diagnostic facilities, such as hormone measurements.^41^, ^42^ Thanks to its simplicity, the Ca/P ratio could be useful in clinical settings characterized by large inflow of patients (eg, general practitioner, emergency room, PHPT screening of osteoporotic patients). In all these conditions Ca/P may perform very well, allowing cost savings and selecting patients as candidates for second‐line diagnostic procedures such as PTH assay and imaging.

## Disclosures

All authors state that they have no conflicts of interest.
